# Dysregulated Adaptive Immunity Is an Early Event in Liver Cirrhosis Preceding Acute-on-Chronic Liver Failure

**DOI:** 10.3389/fimmu.2020.534731

**Published:** 2021-01-26

**Authors:** Sabrina Rueschenbaum, Sandra Ciesek, Alexander Queck, Marek Widera, Katharina Schwarzkopf, Bernhard Brüne, Christoph Welsch, Heiner Wedemeyer, Stefan Zeuzem, Andreas Weigert, Christian M. Lange

**Affiliations:** ^1^ Department of Gastroenterology and Hepatology, University Hospital and University of Duisburg-Essen, Essen, Germany; ^2^ Department of Internal Medicine 1, Goethe-University Hospital Frankfurt, Frankfurt, Germany; ^3^ Institute of Virology, University Hospital Essen, Essen, Germany; ^4^ Faculty of Medicine, Institute of Biochemistry 1, Goethe-University Frankfurt, Frankfurt, Germany

**Keywords:** cellular immunity, T cells, *torque teno* virus, γδ T-cells, systemic inflammation, immune checkpoints

## Abstract

**Introduction:**

Acute-on-chronic liver failure (ACLF) is characterized by high levels of systemic inflammation and parallel suppression of innate immunity, whereas little is known about adaptive immune immunity in ACLF. We therefore aimed to characterize the development of the adaptive immune system during the progression of liver cirrhosis to ACLF. Patients with compensated/stable decompensated liver cirrhosis, acute decompensation of liver cirrhosis, or ACLF were recruited from a prospective cohort study. Comprehensive immunophenotyping was performed using high dimensional flow cytometry. Replication of *Torque teno* (TT) virus was quantified as a marker of immunosuppression. High frequencies of detectable TT virus were observed already in patients with compensated/stable decompensated liver cirrhosis compared to healthy controls (>50% vs. 19%), suggesting relatively early occurrence of immunosuppression in cirrhosis. In line, profoundly reduced numbers of distinct innate and adaptive immune cell populations were observed before ACLF development. These changes were accompanied by parallel upregulation of co-stimulatory (e.g. CD40L, OX40, CD69, GITR, TIM-1) and inhibitory immune checkpoints (e.g. PDPN, PROCR, 2B4, TIGIT) on CD4+ and CD8+ T cells, which again preceded the development of ACLF. On a functional basis, the capacity of CD4+ and CD8+ T cells to produce pro-inflammatory cytokines upon stimulation was strongly diminished in patients with acute decompensation of liver cirrhosis and ACLF.

**Conclusion:**

Impaired innate and—in particular—adaptive cellular immunity occurs relatively early in the pathogenesis of liver cirrhosis and precedes ACLF. This may contribute to the development of ACLF by increasing the risk of infections in patients with liver cirrhosis.

## Introduction

Liver cirrhosis is associated with portal hypertension, impaired intestinal barrier function and intestinal dysbiosis, which result in intestinal translocation of bacteria and bacterial products (so called pathogen-associated molecular patterns, PAMPs) to the portal venous and systemic circulation (1). In addition, stress and death of parenchymal and non-parenchymal liver cells result in the release of danger-associated molecular patterns (DAMPs) such as high mobility group protein B1 (HMGB1), histones, ATP, or urate (1). The exposure of hepatic and systemic immune cells to DAMPs and PAMPs results in production of chemokines, cytokines, growth factors, reactive oxygen-species (ROS), and activation of local and further recruitment of circulating immune cells like Ly-6C+ monocytes which differentiate into macrophages (2, 3). As a consequence, already compensated liver cirrhosis is associated with low-grade chronic systemic inflammation (4). Patients with acute decompensation of liver cirrhosis show significantly higher grades of inflammation, but highest levels of systemic inflammation were consistently observed in patients with acute-on-chronic liver failure (ACLF) (5, 6). ACLF can be triggered by precipitating events such as infections, excessive alcohol exposure, or re-activation of hepatitis B (6, 7). Such precipitating events can fuel cirrhosis-associated systemic inflammation, evidenced by excessive production of inflammatory mediators such as TNF-α, IL-6, or IL-8 (5).

Importantly, liver cirrhosis is not only associated with systemic inflammation, but also with a parallel presence of profound immunosuppression (4). For example, serum concentrations of anti-inflammatory cytokines like IL-10 or IL-1RA are progressively increasing during acute decompensation of cirrhosis or development of ACLF (5). In addition, monocytes of patients with liver cirrhosis are increased in frequency and are considered to display an activated phenotype, but they do not sufficiently respond to further stimulation with LPS, a phenomenon called LPS tolerance (4). This phenomenon is partially based on high expression of the inhibitory tyrosine kinase MERTK on peripheral blood monocytes, which suppresses antibacterial monocyte functions in patients with ACLF (8).

In contrast to the well-known changes in cytokine patterns and innate immune responses during the progression of liver cirrhosis to ACLF, less is known about concomitant changes in the adaptive immune compartment and the functional evolution of immunosuppression. We therefore aimed to perform a comprehensive immunophenotyping study with a focus on cellular adaptive immunity during the progression of liver cirrhosis.

## Patients and Methods

### Study Population

Since August 2013, consecutive patients hospitalized to the University Hospital Frankfurt, Germany, with acute decompensation of liver cirrhosis and/or acute-on-chronic liver failure according to the criteria of the CLIF-EASL consortium (6), were prospectively enrolled in our liver cirrhosis cohort study (9). In 2015, the cohort was extended to patients with compensated/stable decompensated liver cirrhosis, not requiring hospitalization due to decompensation.

The diagnosis of liver cirrhosis was based by combination of clinical, laboratory and imaging findings (ultrasound and transient elastography or share wave elastography) or—rarely—by liver biopsy. Acute decompensation of liver cirrhosis was defined as presence of one of the following criteria: new onset/progression of hepatic encephalopathy graded by West-Haven criteria, gastrointestinal hemorrhage, bacterial infection, or ascites grades II–III, according to the definitions used in the canonic study (6). ACLF was diagnosed according to the ACLF-criteria proposed by the CLIF-EASL consortium (6). Patients not requiring hospitalization due to hepatic decompensation and not meeting criteria of acute decompensation or ACLF were classified as compensated/stable decompensated. Patients were excluded if they were younger than 18 years, in case of pregnancy or breastfeeding, presence of hepatocellular carcinoma (HCC) beyond Milan criteria, presence of infection with human immunodeficiency virus (HIV), or therapy with immunosuppressive agents. All patients who were enrolled in this prospective cohort study until September 2017 with sufficient serum samples were included in the present analysis (TT virus cohort, details below). Comprehensive immunophenotyping analyses was performed in those patients of whom sufficient amounts of live peripheral blood mononuclear cells (PBMCs) were available (details on immunophenotyping below).

All patients provided written informed consent to the study protocol, and the study was approved by the local ethic committee of the University Hospital Frankfurt, Germany.

### Detection and Quantification of TT Virus

For the detection and quantification of TT virus, DNA was isolated from plasma samples using QIAamp DNA Blood Mini Kit (Qiagen) and subjected to real-time PCR analysis using Rotor-Gene Probe PCR Kit (Qiagen, Germany) and a Rotor-Gene-Q instrument (Qiagen). TT virus specific primers and a 5´FAM/3´TAMRA labelled probe as well as a standard plasmid containing TT virus genotype 1a DNA (AB017610.1) were used as described elsewhere (10, 11).

### Immunophenotyping and Flow Cytometry

Immunophenotyping was performed in 22 patients with compensated/stable decompensated liver cirrhosis, 34 patients with acute decompensation of liver cirrhosis and 23 patients with ACLF. Due to limited numbers of PBMCs of individual patients, patients had to be distributed in two non-overlapping subgroups for immunophenotyping, i.e. a primary group to determine frequencies of innate and adaptive immune cell subpopulations (results are shown in **Figures 2** and **3**) and a second group to determine expression of co-stimulatory and inhibitory immune checkpoints on CD4+ T cells and CD8+ T cells (results shown in **Figures 4**–**7**). Of note, data of two and nine patients with compensated and decompensated liver cirrhosis, respectively, who had received a transjugular portosystemic stent shunt, were included in a previously published study (12). In addition, PBMCs of 23 healthy volunteers were analyzed as a reference.

For quantification of frequencies of innate and adaptive immune cells subpopulations, immunophenotyping was performed according to the recommendations of the Human Immunology Project Consortium (13) and according to Wistuba-Hamprecht et al. for of γδ T cells (14). Flow cytometric analysis was performed on peripheral blood mononuclear cells (PBMCs) of patients and healthy controls. PBMCs were isolated by Ficoll density gradient separation (Biocoll Separating Solution, Merck Millipore). Cells were incubated with a human FcR blocking reagent (Miltenyi Biotec) and stained with fluorochrome-coupled antibodies in Brilliant Stain Buffer (BD Horizon™). **SI Table 1** lists all antibodies for general immunophenotyping whereas antibodies listed in **SI Table 2** classify subtypes of γδ T cells. Cellular viability was estimated by 7-AAD incorporation (BioLegend). **SI Table 3** lists all antibodies for analysis of expression of co-stimulatory and inhibitory immune checkpoints on CD4+ T cells and CD8+ T cells with Zombie/Aqua (BioLegend) for liver dead cell staining. Flow cytometric measurements were performed on a BD LSRFortessa™ cytometer. For correct gating fluorescence minus one controls (FMOs), i.e. fully stained cells with the exception of one particular antibody-fluorochrome conjugate, were used to identify cells expressing a given cell surface marker, as described previously (12). Frequency of cell populations were analyzed by using FlowJo V10 software. The gating strategy to determine frequencies of innate and adaptive immune cell subpopulations including γδ T cells has been described previously (12). The gating strategy for quantification of costimulatory and inhibitory immune check points is shown in **SI Figure 1**.

### *Ex Vivo* T Cell Function Assays

For analysis of T cell activation, PBMCs were cultured in RMPI/10%FCS/1% Pen/Strep with Golgi inhibitors 1X Monensin/1X Brefeldin A (both eBioscience) in presence or absence of 32nM PMA and 3,2µM Ionomycin (both Sigma Aldrich) for 6 h. Afterwards, cells were collected and intracellular cytokine staining for IFN-γ and TNF-α was performed using antibodies listed in **SI Table 3** with Zombie/Aqua (BioLegend) for liver dead cell staining.

### Statistical Analyses

Statistical analyses were performed using BiAS, Version 11.06, and GraphPad PRISM5. Group differences were assessed by means of χ^2^ contingency tables or Wilcoxon-Mann-Whitney-U-tests, as appropriate. *P* values < 0.05 were considered to be statistically significant. Associations of outcomes with continuous or dichotomic variables were assessed in linear and logistic regression models, respectively. After univariate analyses, multivariate analyses were performed for significant associations. Multivariate models were obtained by backward selection, using a *P* value >0.15 for removal from the model.

## Results

### Baseline Characteristics of Included Patients

Overall, 131 patients with liver cirrhosis could be analyzed for the presence of TT virus, including 31 patients with compensated/stable decompensated liver cirrhosis, 46 patients with acutely decompensated liver cirrhosis, and 54 patients with ACLF. Of patients with ACLF, 23 (43%), 16 (30%), and 15 (28%) patients had ACLF grades 1, 2, and 3, respectively. Baseline characteristics of the TT virus cohort are shown in **Table 1**. Baseline characteristics of the subgroups of patients in whom immunophenotyping was performed are shown in **SI Table 4**.

**Table 1:** Baseline characteristics of included patients.

	ACLF (n = 54)	acutely decompensated cirrhosis (n = 46)	Compensated/stable decompensated cirrhosis (n = 31)	P-value
Age (years), mean (SD)	55 (10)	55 (10)	57 (11)	n.s.
Male gender, n (%)	40 (74.1)	33 (71.7)	20 (64.5)	n.s.
BMI (kg/m^2^), mean (SD)	28.0 (7.0)	26.1 (6.5)	26.0 (5.6)	n.s.
Etiology of liver disease				
Alcoholic	27 (50)	31 (67)	15 (48)	n.s.
Viral	5 (9)	7 (15)	7 (23)	n.s.
NASH	5 (9)	4 (9)	4 (13)	n.s.
Other	17 (31)	4 (9)	5 (16)	0.01
Leucocytes (/nl), mean (SD)	11.2 (6.4)	8.4 (4.8)	5.5 (2.5)	<0.0001
Hemoglobin (g/dl), mean (SD)	9.4 (1.9)	10.6 (2.6)	10.4 (2.4)	0.004
Platelets (/nl), mean (SD)	116 (78)	139 (98)	105 (59)	n.s.
CRP (mg/dl), mean (SD)	5.1 (4.3)	2.7 (2.9)	1.5 (2.1)	<0.0001
Creatinine (mg/dl), mean (SD)	2.4 (1.3)	1.1 (0.7)	0.9 (0.30)	<0.0001
Bilirubin (mg/dl), mean (SD)	12.1 (12.0)	7.0 (9.1)	4.6 (5.4)	0.004
ALT (U/l), mean (SD)	159 (240)	109 (104)	76 (11)	n.s.
INR, mean (SD)	2.0 (0.8)	1.5 (0.4)	1.4 (0.2)	<0.0001
Albumin (g/dl), mean (SD)	2.9 (0.5)	2.8 (0.4)	3.1 (0.5)	n.s.

ALT, alanine aminotransferase; AST, aspartate aminotransferase; BMI, body mass index; CRP, C-reactive protein; γGT, γ-glutamyl transferase; INR, international normalized ratio.

### TT Virus Is Frequently Detectable and Highly Replicative in Patients With Liver Cirrhosis

**Figure 1:**
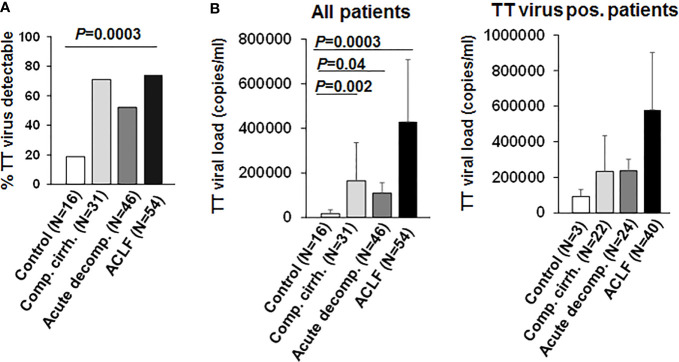
TT virus is frequently detectable in patients with liver cirrhosis. **(A)** Frequencies of detectable TT virus DNA according to the stage of liver disease are shown in comparison to healthy controls. **(B)** Mean quantitative measurement of TT viral load according to the stage of liver disease are shown for all patients (left graph) and for the subgroup of individuals with detectable TT virus (right graph).

*Torque teno virus* (TT virus), is a non-enveloped virus with a circular single-stranded DNA genome, that is highly prevalent in the general population (>95%). Although TT virus replication has not been associated with any human disease, TTV DNA represents a suitable surrogate marker for immune competence (10, 15, 16). Active replication and the magnitude of replication of TT virus are considered to correlate with the extent of immunosuppression in a given patient, e.g. in the setting of medical immunosuppression in organ transplant recipients (15). We therefore performed qualitative and quantitative assessment of TT DNA viral load in our cohort. As shown in **Figure 1**, TT virus was detectable in a significantly higher proportion of patients with liver cirrhosis compared to healthy controls (>50% vs. 19%, *P*=0.0003). In addition, TT viral loads were significantly higher in patients with liver cirrhosis compared to healthy controls (**Figure 1**; *P*<0.05). Of note, frequencies of detectable TT virus were comparable between patients with compensated liver cirrhosis, acute decompensation of liver cirrhosis or ACLF (**Figure 1**). In addition, TT viral load was not significantly (though numerically) higher in patients with ACLF compared to patients with compensated/stable decompensated or acutely decompensated liver cirrhosis (**Figure 1**). Next, we performed logistic regression analyses of factors associated with detectable TT virus in patients with liver cirrhosis. Of note, only the presence of ACLF (P=0.03) and serum creatinine (P=0.02) were associated with detectable TT virus in patients with liver cirrhosis in univariate analysis, whereas in multivariate analysis only serum creatinine (P=0.02, odds ratio=1.60, 95% confidence interval=1.07–2.39) was independently associated with detectable TT virus (**SI Table 5**). Overall, these data suggest a relatively early occurrence of impaired adaptive immune responses to control TT virus in patients with liver cirrhosis, which may progress during development of ACLF.

### Defective Cellular Immunity Occurs Early in Liver Cirrhosis

**Figure 2:**
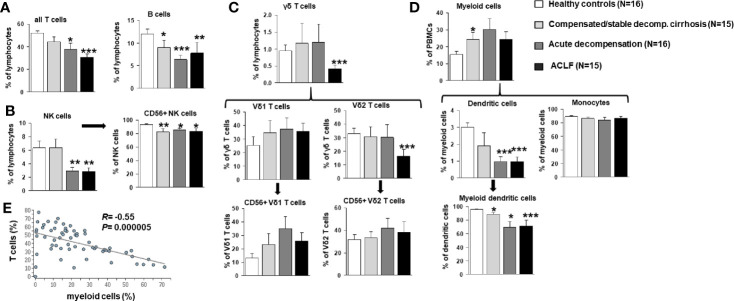
Frequencies of T cells, B cells, NK cells, γδ T cell, and myeloid cells in patients with progressive stages of liver cirrhosis liver cirrhosis vs. healthy controls. **(A)** T and B cell frequencies are represented as frequency of all living lymphocytes. **(B)** NK cells (CD33 positive and CD16 positive) were further classified according to CD56 expression as a marker for cytotoxicity. **(C)** γδ T cells (CD3 positive and γδ TCR positive) were stained for Vδ1, Vδ2 and CD56 to identify major subpopulations. **(D)** Dendritic cells were identified as being CD14 negative and MHCII positive and classified as myeloid dendritic cells if CD11c positive. Cell frequencies are represented as frequency of all living cells for myeloid cells and as frequency of the parent population for all subtypes. **(E)** Correlation between frequencies of T cells and myeloid cells. *P<0.05, **P<0.01, ***P<0.001.

To better understand the evolution of changes of the innate and—in particular—adaptive immune compartments during the progression of liver disease, comprehensive immunophenotyping was performed according to the recommendations of the Human Immunology Project Consortium (13). As shown in **Figure 2A**, a progressive decrease of frequencies of all αβ T cells and of B cells was observed in patients with compensated/stable decompensated *vs.* acutely decompensated liver cirrhosis *vs.* ACLF in comparison to healthy controls. In addition, patients with acutely decompensated liver cirrhosis or ACLF had significantly lower frequencies of NK cells (**Figure 2B**), while only patients with ACLF (but not with decompensated liver cirrhosis) had significantly lower numbers of γδ T cells (**Figure 2C**). The reduced overall frequency of γδ T cells was based on significantly lower numbers of Vd2 γδ T cells, whereas the Vd1 γδ T cell compartment appeared to be expanded in patients with all stages of liver cirrhosis (**Figure 2C**).

As expected, patients with compensated/stable decompensated liver cirrhosis, acutely decompensated liver cirrhosis and ACLF had higher frequencies of myeloid cells in general compared to healthy controls (**Figure 2D**). This observation hold true for classical and non-classical monocytes, whereas reduced frequencies of myeloid dendritic cells were observed in patients with acutely decompensated cirrhosis and ACLF *versus* compensated cirrhosis and healthy controls (**Figure 2D**). Of note, changes in the T cell and myeloid cell compartments correlated strongly (**Figure 2E**).

**Figure 3:**
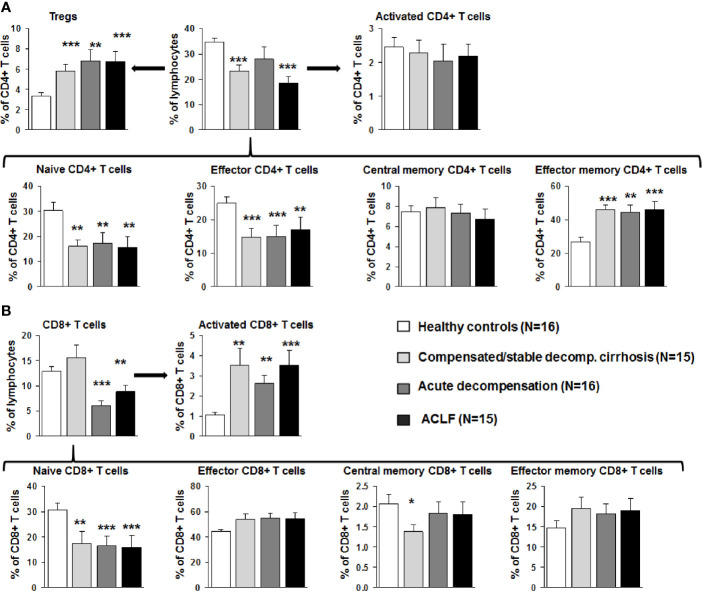
Early changes of the αβ T cell compartment during the course of liver cirrhosis. **(A)** CD4 positive T cells were stained for CD25 and CD127 to identify Tregs (top left), for CD38 and MHCII to assess T cell activation (top right), and for CD45RA and CD197 to further characterize the status of differentiation (bottom panel). Cell frequencies are represented as frequency of all living cells for total CD4 positive T cells (top middle) and as frequency of CD4+ T cells for all subtypes. **(B)** CD8+ positive T cells were stained for CD38 and MHCII to determine activation (top right), and for CD45RA and CD197 to characterize the status of differentiation (bottom panel). Cell frequencies are represented as frequency of all living cells for total CD8 positive T cells (top left) and as frequency of CD8+ T cells for all subtypes. *P<0.05, **P<0.01, ***P<0.001.

Next, detailed subtyping of αβ T cell populations was performed, which are of primary interest in our study. Subtyping revealed reduced numbers of CD4+ T cells at all stages of liver cirrhosis and of CD8+ T cells in patients with acutely decompensated liver cirrhosis and ACLF (**Figure 3**). Changes according to the grade of ACLF, as well as comparison between patients with compensated and stable decompensated cirrhosis, are shown in **SI Figures 2** and **3**. The decrease of CD4+ T cells in acutely decompensated cirrhosis/ACLF was based on reductions of naïve and effector CD4+ T cells, whereas central memory and effector memory CD4+ T cells were not reduced or even increased, respectively (**Figure 3A**). Of note, relative numbers of CD4+ regulatory T cells were significantly higher in patients with compensated/acutely decompensated liver cirrhosis and ACLF in comparison to healthy controls (**Figure 3A**). In the CD8+ T cell compartment, a significant reduction of naïve CD8+ T cells in patients with all stages of liver cirrhosis was observed as well, whereas relative frequencies of effector CD8+ T cells and central memory/central effector T cells were not significantly altered relative to the severity of liver cirrhosis (**Figure 3B**). Importantly, the proportion of activated CD8+ T cells was significantly higher in patients with all stages of liver cirrhosis in comparison to healthy controls (**Figure 3B**). This phenomenon was not directly observed in the CD4+ T cell compartment (**Figure 3A**). However, patients with acutely decompensated liver cirrhosis or ACLF had significantly higher frequencies of central memory Th1 cells, whereas patients with ACLF had lower frequencies of central memory Th17 cells compared to healthy controls (**SI Figure 4**). Comparable, though less pronounced differences were observed for Th1/Th17 effector memory T cell compartment (**SI Figure 4**). In contrast, Th2 cell frequencies were not altered in patients with liver cirrhosis compared to healthy controls (**SI Figure 4**).

### Impaired Adaptive Immune Compartments Are Associated With Replicative TT Virus Infection

We next assessed immune cell frequencies of patients with liver cirrhosis according to the presence of detectable TT virus. As shown in **SI Figure 5**, patients with detectable (i.e. replicative) TT virus in serum revealed significantly lower frequencies of all T cells, naïve CD8+ T cells, central memory CD8+ T cells, and overall CD4+ T cells, whereas frequencies of activated CD8+ T cells were significantly higher in patients with *versus* without detectable TT virus. Of note, Vd2 γδ T cell frequencies were significantly lower in patients with detectable TT virus compared to patients without detectable TT virus as well, whereas no differences in NK cell frequencies were observed (**SI Figure 5**). No differences in myeloid cell populations were observed according to the detectability of TT virus (**SI Figure 5**). Overall, these data suggest a functional relevance of the observed changes of the T cell compartments in patients with liver cirrhosis.

### Profoundly Altered Expression of Co-Stimulatory and Inhibitory Immune Checkpoints on CD4 + and CD8+ T Cells in Patients With Liver Cirrhosis

**Figure 4:**
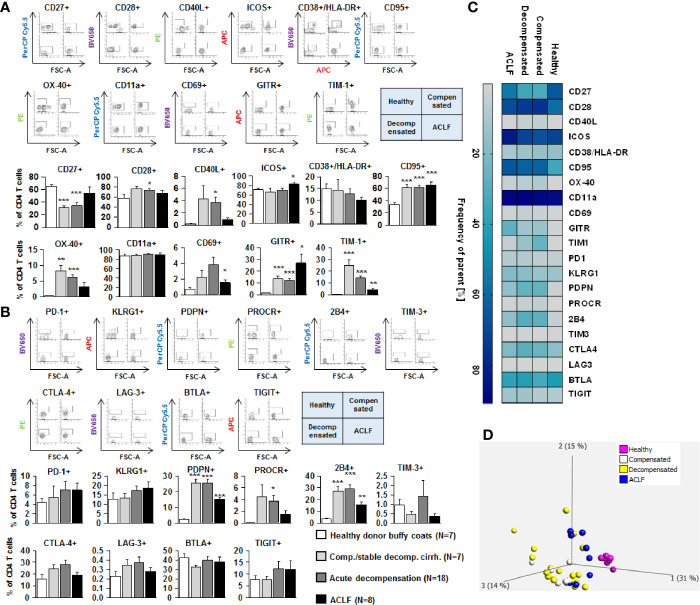
Parallel upregulation of co-stimulatory and inhibitory immune checkpoints on CD4+ effector T cells during the course of liver cirrhosis. Expression of co-stimulatory immune checkpoints, T cells activation markers (CD38/HLA-DR, CD69) as well as of the Fas-ligand CD95 on effector CD4+ T cells are shown in **(A)**, while expression of inhibitory immune checkpoints is shown in **(B)**. Typical examples of dotplots of flow cytometric analysis of healthy controls or patients with compensated liver cirrhosis, decompensated liver cirrhosis or ACLF are shown in the upper panels whereas mean values and standard deviations are shown in graphs in lower panels. **(C)** Heatmap summarizing the mean frequencies of positive cells. **(D)** Principle component analysis of the assessed markers showed distinct clustering of patients with liver cirrhosis versus healthy controls whereas no clear clustering was observed between the stages of liver cirrhosis. Values are displayed as frequency of parent. *P<0.05, **P<0.01, ***P<0.001.

**Figure 5:**
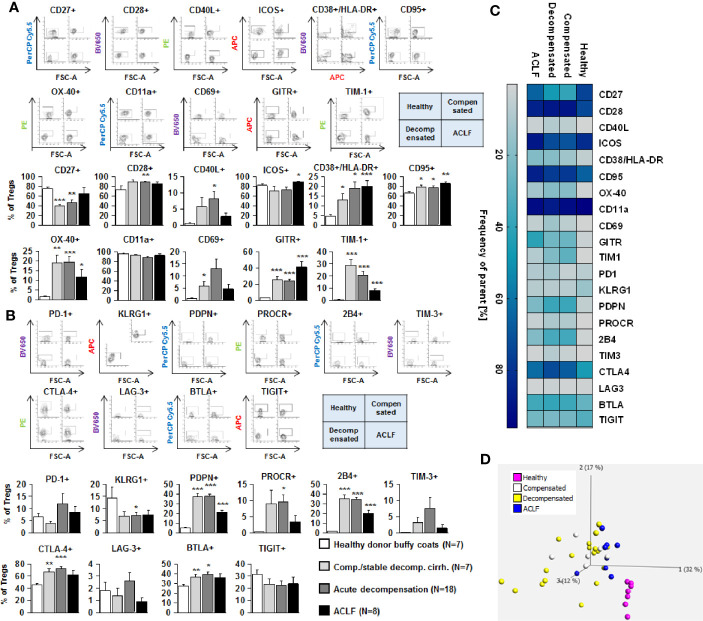
Parallel upregulation of co-stimulatory and inhibitory immune checkpoints on CD4+ regulatory T cells during the course of liver cirrhosis. Expression of co-stimulatory immune checkpoints, T cells activation markers (CD38/HLA-DR, CD69) as well as of the Fas-ligand CD95 on regulatory (CD4+ CD25dim CD127-) T cells are shown in **(A)**, while expression of inhibitory immune checkpoints is shown in **(B)**. Typical examples of dotplots of flow cytometric analysis of healthy controls or patients with compensated liver cirrhosis, decompensated liver cirrhosis or ACLF are shown in the upper panels whereas mean values and standard deviations are shown in graphs in lower panels. **(C)** Heatmap summarizing the mean frequencies of positive cells. **(D)** Principle component analysis of the assessed markers showed distinct clustering of patients with liver cirrhosis versus healthy controls whereas no clear clustering was observed between the stages of liver cirrhosis. Values are displayed as frequency of parent. *P<0.05, **P<0.01, ***P<0.001.

**Figure 6:**
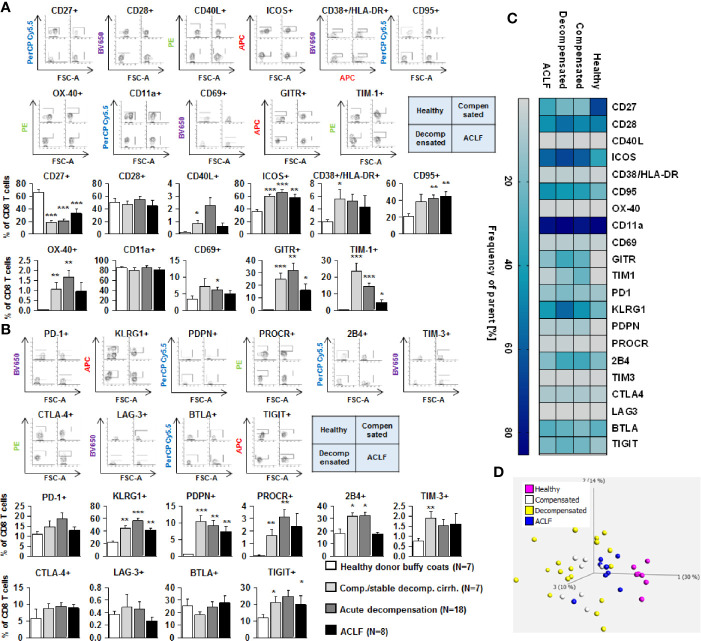
Parallel upregulation of co-stimulatory and inhibitory immune checkpoints on CD8+ effector T cells during the course of liver cirrhosis. Expression of co-stimulatory immune checkpoints, T cells activation markers (CD38/HLA-DR, CD69) as well as of the Fas-ligand CD95 on CD8+ T cells are shown in **(A)**, while expression of inhibitory immune checkpoints is shown in **(B)**. Typical examples of dotplots of flow cytometric analysis of healthy controls or patients with compensated liver cirrhosis, decompensated liver cirrhosis or ACLF are shown in the upper panels whereas mean values and standard deviations are shown in graphs in lower panels. **(C)** Heatmap summarizing the mean frequencies of positive cells. **(D)** Principle component analysis of the assessed markers showed distinct clustering of patients with liver cirrhosis versus healthy controls whereas no clear clustering was observed between the stages of liver cirrhosis. Values are displayed as frequency of parent. *P<0.05, **P<0.01, ***P<0.001.

We next assessed the expression of co-stimulatory and inhibitory immune checkpoints on CD4+ and CD8+ T cells. As shown in **Figures 4A** and **5A**, a number of co-stimulatory molecules, namely CD40L, OX-40, GITR, and TIM-1 were significantly upregulated on both effector (**Figure 4A**) and regulatory CD4+ T cells (**Figure 5A**) in patients with liver cirrhosis compared to healthy controls, as well as the T cell activation markers CD69 and CD38. In contrast, CD28 and CD11a were not significantly altered on CD4+ effector T cells and regulatory T cells, whereas ICOS was only upregulated on CD4+ regulatory T cells of patients with liver cirrhosis. An exceptional expression was observed for the co-stimulatory molecule CD27, which was expressed at significantly lower levels on CD4+ T cells of patients with compensated and acutely decompensated liver cirrhosis. Overall, comparable changes in the expression of co-stimulatory molecules were observed on CD8+ T cells of patients with liver cirrhosis (**Figure 6A**).

Changes of expression of co-stimulatory molecules and T cell activation markers were accompanied by a significant upregulation of the Fas-receptor CD95 on CD4+ T cells and CD8+ T cells (**Figures 4A** **–** **6A**), which promotes T cell apoptosis, as well as by an increased expression of inhibitory immune checkpoints, namely PDPN, KLGR1, PROCR, and 2B4 on CD4+ effector T cells, CD4+ regulatory T cells and CD8+ T cells, whereas upregulation of the inhibitory immune checkpoints PD-1, CTLA4, BTLA, TIM-3, LAG-3, and TIGIT in patients with liver cirrhosis was more variable (**Figures 4B** **–** **6B**). Further heatmap and principal component analyses revealed that maximal changes in the expression of co-stimulatory and inhibitory immune checkpoints relative to healthy controls were observed in patients with compensated/stable decompensated and acutely decompensated cirrhosis, while in patients with ACLF expression levels of these molecules declined to intermediate levels between healthy controls and patients with compensated/stable decompensated and acutely decompensated cirrhosis. Changes according to the grade of ACLF, as well as comparison between patients with compensated and stable decompensated cirrhosis, are shown in **SI Figures 2** and **3**.

### Weak Correlation Between Systemic Inflammation and Changes in the Adaptive Immune System

**Figure 7:**
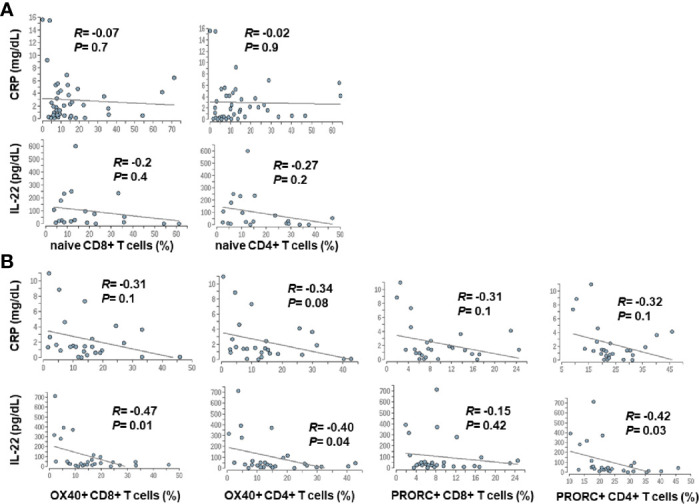
Correlation between markers of systemic inflammation and changes in the adaptive immune system in patients with liver cirrhosis. Serum concentrations of CRP and IL-22, which are well established markers of systemic inflammation in cirrhosis (9), were correlated with frequencies of naïve CD4+ and CD8+ T cells **(A)**, was well as with the expression of selected co-stimulatory (OX40) and inhibitory (PRORC) immune checkpoints **(B)**.

Since advanced liver cirrhosis and in particular ACLF are characterized by profound systemic inflammation, we performed a correlation between markers of systemic inflammation [CRP and IL-22 (9)] and selected features of the adaptive immune system. Weak, non-significant correlations between systemic inflammation and the number of naïve CD4+ and CD8+ T cells were observed, whereas a moderate association between the extend of systemic inflammation and expression of co-stimulatory and inhibitory immune checkpoints (OX40, PRORC) on CD4+ and CD8+ T cells was observed (**Figure 7**).

### Inappropriate Function of Effector T Cells of Patients With Liver Cirrhosis

**Figure 8:**
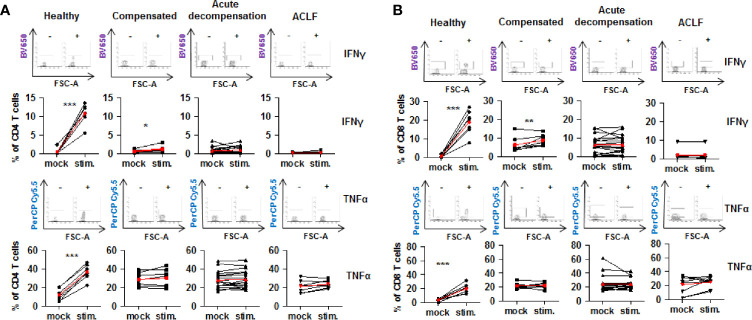
Impaired on-demand production of pro-inflammatory cytokines in CD4+ and CD8+ T cells of patients with liver cirrhosis. CD4+ T cells **(A)** and CD8+ T cells **(B)** from healthy controls are patients with compensated/stable decompensated liver cirrhosis, acute decompensation of liver cirrhosis, or ACLF were stimulated ex vivo with PMA/Ionomycin for 6 h. Production of IFN-γ and TNF-α at baseline before stimulation and after stimulation were assessed by flow cytometry. Typical examples of dotplots of flow cytometric analysis of healthy controls or patients with compensated liver cirrhosis, decompensated liver cirrhosis or ACLF are shown in the upper panels whereas mean values and standard deviations are shown in graphs in lower panels. Red bars indicate mean increase of pro-inflammatory cytokine production from baseline to 6 h after stimulation. *P<0.05, **P<0.01, ***P<0.001.

Collectively, the above described findings reveal a parallel increased expression of co-stimulatory and inhibitory immune checkpoints on CD4+ and CD8+ T cells of patients with liver cirrhosis. To understand the functional consequences of these observations, live T cells of patients with liver cirrhosis or healthy controls were stimulated *ex vivo* with PMA/ionomycin. Of note, baseline expression of IFN-γ and TNF-α appeared to be higher in CD4+ and CD8+ T cells of patients with all stages of liver cirrhosis compared to healthy controls. As shown in **Figure 8**, stimulation with PMA/ionomycin of CD4+ and CD8+ T cells from healthy controls resulted in a strong and significant increase of production of IFN-γ and TNF-α, and in an attenuated but still significant increase of IFN-γ, but not of TNF-α of CD4+ and CD8+ T cells of patients with compensated/stable decompensated liver cirrhosis. In contrast, CD4+ and CD8+ T cells of patients with acutely decompensated cirrhosis or with ACLF did not respond to stimulation with PMA/ionomycin with induction of IFN-γ and TNF-α.

## Discussion

The present comprehensive immunophenotyping study suggests a relatively early occurrence of impaired cellular immune responses during the course or liver cirrhosis, indicated by strongly reduced numbers of important cell populations of the adaptive and innate immune system like CD4+ T cells, CD8+ T cells, B cells, NK cells, and dendritic cells, which are accompanied by a parallel induction of co-stimulatory and inhibitory immune checkpoints on CD4+ and CD8+ T cells and a lost capacity to induce pro-inflammatory cytokine production. Most of the observed changes were already evident in patients with compensated/stable decompensated liver cirrhosis and were fully developed in patients with acutely decompensated liver cirrhosis, while ACLF was associated with only few additional changes in immune cell frequencies like a restricted compartment of V2 γδ T cells and declining levels of immune checkpoint expression on αβ T cells. Hence, impaired cellular immune compartments are preceding ACLF and may contribute to the pathogenesis of ACLF. Furthermore, the high frequency of active replication of TT virus observed in our cohort of patients with liver cirrhosis supports the functional relevance of the altered cellular immune compartments.

The impaired immune cell compartments observed in our study develop in parallel to a progressive systemic inflammatory response, which is evidenced by progressively increased levels of pro-inflammatory mediators such as cytokines, chemokines, or eicosanoids in patients with compensated liver cirrhosis *versus* acutely decompensated cirrhosis *versus* ACLF (1, 5, 17). Already compensated liver cirrhosis is considered as a disorder accompanied with low-grade systemic inflammation, which promotes symptoms of liver cirrhosis such as fatigue or frailty (4). During the progression of liver cirrhosis to decompensated liver cirrhosis or ACLF, systemic inflammation augments progressively to levels which are sufficient to induce organ failures and ultimately death (1, 4, 5, 18). Importantly, systemic inflammation in patients with liver cirrhosis is paralleled by a state of immunosuppression, resulting in a high risk of development and adverse outcome of infections in liver cirrhosis and—in particular—in ACLF. This is evidenced by a kinetic of anti-inflammatory cytokines such as IL-10 or IL-1ra which completely parallels the increasing production of pro-inflammatory cytokines during the progression of liver cirrhosis to ACLF (5). The parallel increased expression of co-stimulatory and inhibitory immune checkpoints on CD4+ and CD8+ T cells observed in our study resembles the above described co-existence of upregulated pro- and anti-inflammatory cytokines and innate immune pathways which result in an ineffective host defense despite taking the hazards of inflammation-induced organ failures. Indeed, CD4+ and CD8+ T cells of patients with liver cirrhosis in our study showed high expression of pro-inflammatory cytokines at baseline which may contribute to inflammation-induced organ failures, but a lacking on-demand increase in response to further stimulation. Of note, expression of co-stimulatory and inhibitory immune checkpoints on T cells peaked in patients with compensated and acutely decompensated cirrhosis and diminished in patients with ACLF, which likely reflects extended exhaustion of adaptive immunity in patients with ACLF.

Collectively, systemic inflammation, production of inhibitory cytokines and impaired innate and adaptive cellular immune responses evolve in parallel in patients with liver cirrhosis. In this regard, liver cirrhosis could be considered as a disorder which is characterized by an impaired resolution of inflammation. The concept of resolution of inflammation includes appropriate removal of inflammatory mediators, appropriate termination and clearance of secondary anti-inflammatory cells and mediators, as well as an adequate tissue repair (19). All these features are lacking in patients with liver cirrhosis. Of note, it has been shown that an inadequate resolution of inflammation results in subsequent dysfunctional adaptive immunity characterized by T cell fate, activation of T cells, and impaired T cell function (19). The results of our study would be in line with a concept of impaired T cell immunity as a result of inappropriate resolution of inflammation, as we have indeed observed higher frequencies of activated T cells, of effector memory CD4+ T cells, as well as of induction of co-stimulatory immune checkpoints, which are however accompanied by impaired high levels of inhibitory immune checkpoints, lacking induction of cytokines upon stimulation of CD4+ and CD8+ T cells and impaired control of TT virus replication in patients with liver cirrhosis.

Of particular importance, the here observed impaired adaptive immune compartment evolves relatively early in the progress of liver cirrhosis, is almost completely established in acutely decompensated cirrhosis and appears to precede ACLF. However, it is important to note that we applied the concept of acute decompensation of liver cirrhosis in our study, which was recently introduced by the Cliff consortium and which differs from the classical concept of decompensation (6). The finding of reduced T cell numbers in patients with early stages of liver cirrhosis is in line with previous studies in patients with chronic hepatitis C and other causes of liver cirrhosis (20–22). Although our study is of associative nature and does not allow causal conclusions, one may speculate that impaired adaptive immune responses may promote the development and adverse outcome of infections, as infections are one of the most frequent causes of ACLF (6). Furthermore, infections are frequent complications of ACLF, and infection-triggered ACLF is associated with a particular poor outcome (23, 24). Collectively, these clinical data and the results of our immunophenotyping study support further research to assess whether strategies to reset the adaptive immune system to a naïve state to enable an appropriate response to pathogens (i.e. a strategy to promote resolution of inflammation), would be of benefit in the prevention and treatment of ACLF.

Our study has important limitations. First of all, we were only able to analyze immune cells in the peripheral blood of patients with liver cirrhosis, because liver biopsies in patients with advanced liver disease are rarely performed at our department. Hence, the here described immune phenotypes may differ in other relevant immunological compartment of patients with liver cirrhosis, in particular in the liver and intestines (25). Furthermore, we were not able to analyze antigen-specific T cells due to limited cell numbers available for immunophenotyping. Finally, the different stages of liver cirrhosis were analyzed in different patients and not during the progression of cirrhosis in the same individual, which—however—is not an uncommon study design.

Nevertheless, our study provides evidence of pronounced alterations of innate and—in particular—adaptive cellular immunity which precedes ACLF and may contribute to the pathogenesis of ACLF by increasing the risk of infections in patients with liver cirrhosis.

## Data Availability Statement

All datasets generated for this study are included in the article/**Supplementary Material**.

## Ethics Statement

The studies involving human participants were reviewed and approved by Ethics committee of the faculty of medicine, University Hospital Frankfurt. The patients/participants provided their written informed consent to participate in this study.

## Author Contributions

The authors have contributed to the manuscript by planning the study (SR, CL), collecting the data (SR, SC, AQ, MW, KS, CW, AW, CL), analysis and interpretation of data (SR, SC, AQ, MW, BB, CW, HW, SZ, AW, CL), and preparation and revision of the manuscript (all authors). All authors contributed to the article and approved the submitted version.

## Funding

This study was supported by the Deutsche Forschungsgemeinschaft (LA 2806/2-1 and LA 2806/5-1 to CL).

## Conflict of Interest

The authors declare that the research was conducted in the absence of any commercial or financial relationships that could be construed as a potential conflict of interest.
