# Supplementary material for: Dysregulated Adaptive Immunity Is an Early Event in Liver Cirrhosis Preceding Acute-on-Chronic Liver Failure
Source: Front Immunol. 2021 Jan 26;11:534731. doi: 10.3389/fimmu.2020.534731 (PMC7870861; doi:10.3389/fimmu.2020.534731)
Supplement: Supplementary file 1 [file Table_1.docx]

**SI Information_Rüschenbaum et al.**

**SI Table 1: Antibodies for general immunophenotyping, according to the recommendations of the Human Immunology Project Consortium (Maecker et al.; (1))**

| **Target** | **Fluorochrome** | **Clone** | **Art. Nr.** | **Company** |
| --- | --- | --- | --- | --- |
| CD3 | **FITC** | UCHT1 | 300452 | Biolegend |
| CD4 | **PE-CF594** | RPA-T4 | 300548 | Biolegend |
| CD8 | **BV650** | RPA-T8 | 301042 | Biolegend |
| CD11c | **PE-CF594** | Bu15 | 337228 | Biolegend |
| CD14 | **PerCP Cy 5.5** | HCD14 | 325622 | Biolegend |
| CD16 | **BV650** | 3G8 | 302042 | Biolegend |
| CD19 | **APC/Fire750** | SJ25C1 | 363030 | Biolegend |
| CD25 | **BV421** | M-A251 | 356114 | Biolegend |
| CD33 | **BV510** | WM53 | 303422 | Biolegend |
| CD38 | **APC** | HB-7 | 356606 | Biolegend |
| CD45RA | **BV711** | HI100 | 304138 | Biolegend |
| CD45RO | **AF700** | UCHL1 | 304218 | Biolegend |
| CD56 | **BV605** | 5.1H11 | 362538 | Biolegend |
| CD85g | **PE** | 17G10.2 | 326408 | Biolegend |
| CD127 | **BV605** | A019D5 | 351334 | Biolegend |
| CD183 | **PerCP Cy 5.5** | G025H7 | 353714 | Biolegend |
| CD196 | **PE** | G034E3 | 353410 | Biolegend |
| CD197 | **PE-Cy7** | G043H7 | 353226 | Biolegend |
| HLA-DR | **APC/Fire750** | L243 | 307658 | Biolegend |
| 7-AAD | **PE-Cy5** |  | 420404 | Biolegend |

**SI Table 2: Antibodies for phenotyping of** γδ T cells**, according to the recommendations of Wistuba-Hamprecht et al. (2).**

| **Target** | **Fluorochrome** | **Clone** | **Art. Nr.** | **Company** |
| --- | --- | --- | --- | --- |
| CD3 | **AF700** | UCHT1 | 300424 | Biolegend |
| CD4 | **PE-CF594** | RPA-T4 | 300548 | Biolegend |
| CD8 | **BV650** | RPA-T8 | 301042 | Biolegend |
| CD16 | **BV421** | 3G8 | 302038 | Biolegend |
| CD27 | **PerCP Cy 5.5** | O323 | 302820 | Biolegend |
| CD28 | **APC** | CD28.2 | 302912 | Biolegend |
| CD45RA | **BV711** | HI100 | 304138 | Biolegend |
| CD56 | **BV605** | 5.1H11 | 362538 | Biolegend |
| HLA-DR | **APC/Fire750** | L243 | 307658 | Biolegend |
| γδ-TCR | **PE-Cy7** | 11F2 | 655410 | BD Biosciences |
| Vδ1-TCR | **FITC** | TS8.2 | TCR2730 | Thermo Fisher |
| Vδ2-TCR | **PE** | B6 | 331408 | Biolegend |
| 7-AAD | **PE-Cy5** |  | 420404 | Biolegend |

**SI Table 3: Antibodies for quantification of co-stimulatory and inhibitory immune checkpoints on CD4+ and CD8+ T cells.**

| **Target** | **Fluorochrome** | **Clone** | **Art. Nr.** | **Company** |
| --- | --- | --- | --- | --- |
| CD3 | **FITC** | UCHT1 | 300452 | BioLegend |
| CD4 | **AF700** | RPA-T4 | 300526 | BioLegend |
| CD8 | **APC/Fire750** | RPA-T8 | 301066 | BioLegend |
| CD25 | **BV421** | M-A251 | 356114 | BioLegend |
| CD45RA | **PE-CF59** | SJ25C1 | 304146 | BioLegend |
| CD127 | **BV605** | A019D5 | 351334 | BioLegend |
| CD197 | **PE-Cy7** | G043H7 | 353226 | BioLegend |
| CD11a | **PerCP Cy 5.5** | TS2/4 | 350614 | BioLegend |
| CD27 | **PerCP Cy 5.5** | O323 | 302820 | BioLegend |
| CD28 | **BV650** | CD28.2 | 302946 | BioLegend |
| CD38 | **APC** | HB-7 | 356606 | BioLegend |
| CD69 | **BV650** | FN50 | 310934 | BioLegend |
| CD95 | **PerCP Cy 5.5** | DX2 | 305630 | BioLegend |
| CD134 (= OX-40) | **PE** | Ber-ACT35 | 350004 | BioLegend |
| CD152 (= CTLA-4) | **PE** | BNI3 | 369604 | BioLegend |
| CD154 (= CD40L) | **PE** | 24-31 | 310806 | BioLegend |
| CD201 (= PROCR) | **PE** | RCR-401 | 351904 | BioLegend |
| CD223 (= LAG-3) | **BV650** | 11C3C65 | 369316 | BioLegend |
| CD244 (= 2B4) | **PerCP Cy 5.5** | C1.7 | 329516 | BioLegend |
| CD272 (= BTLA) | **PerCP Cy 5.5** | MIH26 | 344514 | BioLegend |
| CD278 (= ICOS) | **APC** | C398.4A | 313510 | BioLegend |
| CD279 (= PD-1) | **BV650** | EH12.2H7 | 329950 | BioLegend |
| CD357 (= GITR) | **APC** | 108-17 | 371206 | BioLegend |
| CD365 (= TIM-1) | **PE** | 1D12 | 353904 | BioLegend |
| CD366 (= TIM-3) | **BV650** | F38-2E2 | 345028 | BioLegend |
| HLA-DR | **BV650** | L243 | 307650 | BioLegend |
| KLRG1 | **APC** | SA231A2 | 367716 | BioLegend |
| PDPN | **PerCP Cy 5.5** | NC-08 | 337012 | BioLegend |
| TIGIT | **APC** | A15153G | 372706 | BioLegend |
| IFNγ | **BV650** | 4S.B3 | 502538 | BioLegend |
| TNFα | **PerCP Cy 5.5** | Mab11 | 502962 | BioLegend |

SI Table 4: Baseline characteristics of patients included in the immunophenotyping cohort.

|  | **ACLF** | **Acute decompensation** | **compensated/stable decompensated cirrhosis** | **P-value** |
| --- | --- | --- | --- | --- |
| **Subgroup 1, frequencies of innate and adaptive immune cell subpopulations** | | | | |
| Number | 15 | 16 | 15 |  |
| Age (years), mean (SD) | 57 (12) | 59 (8) | 57 (12) | n.s. |
| Male gender, n (%) | 13 (87.0) | 10 (62.5) | 9 (60.0) | n.s. |
| BMI (kg/m^2^), mean (SD) | 28.1 (7.1) | 26.6 (7.6) | 25.3 (5.3) | n.s. |
| Leucocytes (/nl), mean (SD) | 11.6 (6.1) | 7.5 (3.3) | 5.4 (2.2) | <0.001 |
| Hemoglobin (g/dl), mean (SD) | 9.2 (1.7) | 10.8 (2.7) | 10.6 (2.7) | 0.05 |
| Platelets (/nl), mean (SD) | 103 (67) | 130 (93) | 132 (79) | n.s. |
| CRP (mg/dl), mean (SD) | 3.9 (3.8) | 2.7 (3.7) | 2.0 (3.0) | 0.06 |
| Creatinine (mg/dl), mean (SD) | 2.5 (1.3) | 1.2 (0.5) | 1.0 (0.30) | <0.0001 |
| Bilirubin (mg/dl), mean (SD) | 15.7 (13.4) | 6.7 (11.7) | 2.8 (0.8) | 0.002 |
| ALT (U/l), mean (SD) | 143 (129) | 89 (93) | 60 (22) | n.s. |
| INR, mean (SD) | 2.3 (1.0) | 1.5 (0.4) | 1.4 (0.3) | 0.002 |
| Albumin (g/dl), mean (SD) | 2.7 (0.6) | 2.9 (0.4) | 3.3 (0.7) | 0.1 |
| **Subgroup 2, immune checkpoints** | | | | |
| Number | 8 | 18 | 7 |  |
| Age (years), mean (SD) | 57 (7) | 52 (12) | 51 (11) | n.s. |
| Male gender, n (%) | 5 (62.5) | 12 (66.7) | 5 (71.4) | n.s. |
| BMI (kg/m^2^), mean (SD) | 30.1 (5.1) | 26.0 (7.1) | 25.4 (6.8) | 0.04 |
| Leucocytes (/nl), mean (SD) | 8.6 (8.5) | 7.7 (7.3) | 6.7 (4.1) | n.s. |
| Hemoglobin (g/dl), mean (SD) | 8.9 (2.4) | 9.8 (3.0) | 10.4 (2.1) | 0.1 |
| Platelets (/nl), mean (SD) | 82 (58) | 127 (97) | 98 (56) | n.s. |
| CRP (mg/dl), mean (SD) | 4.8 (4.1) | 1.8 (1.7) | 0.9 (0.7) | 0.02 |
| Creatinine (mg/dl), mean (SD) | 2.7 (1.4) | 1.0 (0.4) | 0.8 (0.30) | 0.0001 |
| Bilirubin (mg/dl), mean (SD) | 10.8 (11) | 5.6 (6.2) | 4.5 (4.6) | n.s. |
| ALT (U/l), mean (SD) | 46 (28) | 89 (107) | 35 (33) | n.s. |
| INR, mean (SD) | 1.8 (0.5) | 1.6 (0.6) | 1.3 (0.3) | n.s. |
| Albumin (g/dl), mean (SD) | 2.7 (0.5) | 2.6 (0.7) | 3.1 (0.6) | n.s. |

ALT, alanine aminotransferase; AST, aspartate aminotransferase; BMI, body mass index; CRP, C-reactive protein; γGT, γ-glutamyl transferase; INR, international normalized ratio;

SI Table 5. Logistic regression analyses of factors associated with detectable TT virus at baseline.

INR, international normalized ratio.

|  | **Univariate** | | **Multivariate** | |
| --- | --- | --- | --- | --- |
|  | **OR (95% CI)** | ***P* value** | **OR (95% CI)** | ***P* value** |
| Age | 1.02 (0.98-1.06) | 0.3 |  |  |
| Female sex | 0.57 (0.26-1.25) | 0.16 |  |  |
| Presence of ACLV | 2.36 (1.09-5.14) | 0.03 |  |  |
| Leucocytes /nL | 0.98 (0.91-1.04) | 0.5 |  |  |
| Hemoglobin (g/dL) | 0.91 (0.78-1.07) | 0.3 |  |  |
| Platelets /nL | 1.00 (0.99-1.00) | 0.13 |  |  |
| Creatinine (mg/dL) | 1.60 (1.07-2.39) | 0.02 | 1.60 (1.07-2.39) | 0.02 |
| Bilirubin (mg/dL) | 0.99 (0.96-1.03) | 0.8 |  |  |
| INR | 1.13 (0.63-2.02) | 0.7 |  |  |

**SI Figures**

**SI Figure 1. Gating strategy to quantify costimulatory and inhibitory immune checkpoints on CD4+ and CD8+ T cells.** It starts with gating on all myeloid and lymphocyte subsets, thereby termed “Leukocytes” (1) followed by singlet gating (2 and 3). In order to include only “All living T-cells”, live-dead staining Zombie Aqua vs. CD3 was applied (4). This is followed by identification of CD4+ and CD8+ T cell subsets (5). Regulatory T-cells (Tregs) are characterized as being CD25dim CD127 negative T cells (6). Within all three groups of CD8+, CD4+ and regulatory T cells co-stimulatory and inhibitory marker expression was analyzed based on fluorescence signals for PercP Cy5.5 (e.g. CD27), for BV650 (e.g. CD28), for APS (e.g. ICOS) and for PE (e.g. CD40L) according to the antibody list in SI table 3 (7-10).

**SI Figure 2. Comparative analysis of compensated versus stable decompensated cirrhosis.** Exemplarily, data are shown for differences in TT viral load (A), frequencies of Tregs, naive CD4+ and CD8+ T cells (B), as well as the expression pattern of OX-40 and PDPN on CD4+, Tregs and CD8+ T cells (C).

**SI Figure 3. Comparative analysis according to the grade of ACLF.** Exemplarily, data are shown for differences in TT viral load (A), frequencies of Tregs, naive CD4+ and CD8+ T cells (B), as well as the expression pattern of OX-40 and PDPN on CD4+, Tregs and CD8+ T cells (C).

**SI Figure 4. Phenotyping of TH cell subpopulations of CD4 memory T cells.** TH cells were identified according to the expression of CD183 (Th1 cells are positive) and CD196 (Th17 cells are positive, whereas Th2 cells are negative for both markers), according to Maecker et al (1). Cell frequencies are represented in frequency of the parent population for all cell types. **P*<0.05, ***P*<0.01, ****P*<0.001.

**SI Figure 5. Restraint T cell compartments are associated with detectable TT virus in patients with liver cirrhosis.** Phenotyping of immune cell populations in patients with liver cirrhosis was performed as described in Figures 2-3 in the main manuscript and presented according to the detectability of TT virus.

**SI References**

1. Maecker HT, McCoy JP, Nussenblatt R. Standardizing immunophenotyping for the Human Immunology Project. Nat Rev Immunol 2012;12:191-200.

2. Wistuba-Hamprecht K, Pawelec G, Derhovanessian E. OMIP-020: phenotypic characterization of human gammadelta T-cells by multicolor flow cytometry. Cytometry A 2014;85:522-524.
